# Old cemeteries help to protect endangered and protected vascular plant species in the Right–Bank of Dnipro Grass Steppe District, southern Ukraine

**DOI:** 10.3897/BDJ.13.e176357

**Published:** 2025-12-02

**Authors:** Nadiia Skobel, Ivan Moysiyenko

**Affiliations:** 1 Kherson State University, Kherson, Ukraine Kherson State University Kherson Ukraine; 2 F. Falz-Fein Biosphere Reserve “Askania Nova”, Kyiv, Ukraine F. Falz-Fein Biosphere Reserve “Askania Nova” Kyiv Ukraine; 3 M. Kholodnyi`s Institute of botany NAS Ukraine, Kyiv, Ukraine M. Kholodnyi`s Institute of botany NAS Ukraine Kyiv Ukraine

**Keywords:** biodiversity, floristic richness, protected species, grasslands, Red Data Book of Ukraine, Resolution 6 of the Bern Convention, species-area relationships

## Abstract

**Background:**

The dataset contains records of vascular plant species occurrences and distribution in 39 old cemeteries of the Right–Bank Dnipro Grass Steppe District (southern Ukraine). It documents 635 vascular plant species. Notably, the high vascular plant diversity of old cemeteries includes 58 protected species (10.4% of the total recorded flora) with varying conservation statuses. The dataset underscores the conservation significance of old cemeteries in southern Ukraine by documenting the presence of protected species. Old cemeteries contribute to the conservation of endangered and protected vascular plant species outside legally protected steppe areas. Therefore, where they are abundant, they may play an important role in the future restitution of the European and Eurasian steppes.

**New information:**

The dataset contains 6,923 occurrences of vascular plants (635 species) recorded in 2023-2024 in 39 old cemeteries of the Right–Bank Dnipro Grass Steppe District. The dataset includes information on 427 occurrences of 58 protected species of particular conservation interest mentioned in Resolution 6 of the Bern Convention as well as in the Red Data Book of Ukraine and regional Red Lists. This dataset highlights the significance of old cemeteries as pivotal refugia for vascular plant biodiversity within southern Ukraine. The high number of endangered and protected vascular plant species present in old cemeteries provides a critical opportunity for future conservation efforts, thereby contributing directly to biodiversity protection objectives.

## Introduction

Anthropogenic activities have led to a significant loss of natural habitats over the last few centuries worldwide (Löki et al. 2019, Radić and Gavrilović 2020) and in the Ukraine (Burkovskyi et al. 2013). Steppes once occupied about 40% of the territory of Ukraine (Korotchenko and Peregrym 2012), while, today, steppe remnants have survived in only 1-3% of the natural and semi-natural steppes (Burkovskyi et al. 2013, Korotchenko and Peregrym 2012).

The steppe zone of Ukraine faces acute anthropogenic pressure where intensive agriculture and land-use practices, driven by the nutrient-rich and easily cultivated chornozem soils, as well as extensive steppe areas located in favourable topographic conditions and secondly resulted from exceptionally easy terms for agricultural exploitation with minimal need of drainage or tree removal (Korotchenko and Peregrym 2012, Burkovskyi et al. 2013), have largely eliminated the majority of natural steppe flora threatening numerous protected and characteristic steppe species.

As a result, the steppes have been almost completely destroyed and are preserved today mainly in protected areas and in habitats unsuitable for agriculture (ravines, gullies, river terraces and sea cliffs) (Burkovskyi et al. 2013). Consequently, the profound degradation and fragmentation of the steppe necessitate a quantitative assessment of protected species abundance and the viability of remnant populations within these small habitat islands. The ultimate goal is the designation of valuable sites as Nature Conservation Areas. Such formalisation is essential to prevent their continued destruction through agricultural encroachment or land recultivation.

The problem of biodiversity conservation is recognised as one of the global problems of humanity and is a priority at the state level (Chusova et al. 2022). This is evidenced by the close attention paid to this issue by international nature conservation organisations operating under the auspices of UNESCO, IUCN etc. (Kuzemko 2018, Vasyliuk et al. 2022).

Recent studies have demonstrated the high importance of habitat islands of anthropogenic origin, such as burial mounds (kurgans) (Moysiyenko and Sudnik-Wójcikowska 2006a, Moysiyenko and Sudnik-Wójcikowska 2006b, Sudnik-Wójcikowska and Moysiyenko 2006, Moysiyenko and Sudnik-Wójcikowska 2008, Sudnik-Wójcikowska and Moysiyenko 2008, Moysiyenko and Sudnik-Wójcikowska 2009, Moysiyenko and Sudnik-Wójcikowska 2010, Sudnik-Wójcikowska and Moysiyenko 2010, Sudnik-Wójcikowska et al. 2011, Sudnik-Wójcikowska and Moysiyenko 2011a, Sudnik-Wójcikowska and Moysiyenko 2011b, Sudnik-Wójcikowska and Moysiyenko 2011c, Sudnik-Wójcikowska and Moysiyenko 2012, Moysiyenko et al. 2014, Deák et al. 2016a, Deák et al. 2016b, Deák et al. 2018, Valkó et al. 2018, Deák et al. 2019, Deák et al. 2020, Moysiyenko et al. 2023, Deák et al. 2024), old settlements (Dayneko 2020, Dayneko et al. 2020, Dayneko et al. 2023) and old cemeteries (Molnár et al. 2018, Barrett and Barrett 2001, Seaton et al. 2015, Czarna 2016, Kowarik et al. 2016, Čanády and Mošanský 2017, Karger et al. 2017, Molnár et al. 2018a, Löki et al. 2019, Nowińska et al. 2020, Itescu and Jeschke 2024), as sites for the preservation of biodiversity, under extensive land use and abandonment (Löki et al. 2019). In our study, we define old cemeteries as cultural heritage sites established more than 100 years ago (Moysiyenko et al. 2021).

We found that the presence of characteristic steppe species, such as *Festuca valesiaca* agg., *Koeleria macrantha*, *Stipa capillata* and a high proportion of natural non-synanthropic and protected species, compared to invasive species, indicates a comparatively high degree of preservation of steppe flora in the old cemeteries. According to the first studies, the flora of the Right–Bank of Dnipro Grass Steppe District includes more than 650 species of vascular plants and 60 protected species of vascular plants (Moysiyenko et al. 2021, Skobel and Moysiyenko 2021, Skobel et al. 2023, Skobel et al. 2024).

The sacred status of old cemeteries, protected by cultural reverence (Moysiyenko et al. 2021), has allowed the long-term persistence of steppe flora, positioning them as indispensable subjects for conservation research and future protective designation.

### The aims that were set for this study

**The aim** of this research is to evaluate the species richness in old cemeteries of the Right–Bank Dnipro Grass Steppe District, analyse the presence of the protected vascular plant species and propose the potential designation of Emerald Network Sites for these areas.

## Project description

### Title

IAVS Special grant to support the research of Ukrainian members: "Plant diversity and species–area relationships modelling of steppe enclaves within old cemeteries of Northern Prychornomoria region (Northern Black Sea Region) of Southern Ukraine", with the support of the programme-targeted of the National Academy of Sciences of Ukraine, 0125U000701 ("Development and use of methodologies and algorithms for assessing the impact of military actions on the phytodiversity of natural ecosystems in Ukraine to determine their losses, restorative and adaptive potential").

## Sampling methods

### Study extent

In our study, we define old cemeteries as cultural heritage sites established more than 100 years ago (Moysiyenko et al. 2021). We sampled 39 old cemeteries located within the Right-Bank Dnipro Grass Steppe District (the western Bank of Ukraine, southern Ukraine). Territory according to the Geobotanical zoning of Ukraine and adjacent areas (Didukh and Shelyag-Sosonko 2003), i.e. Dnipopetrovsk, Mykolaiv and Odesa administrative Regions (47.96-44.80° N, 34.80-28.96 °E).

**Figure 1.**
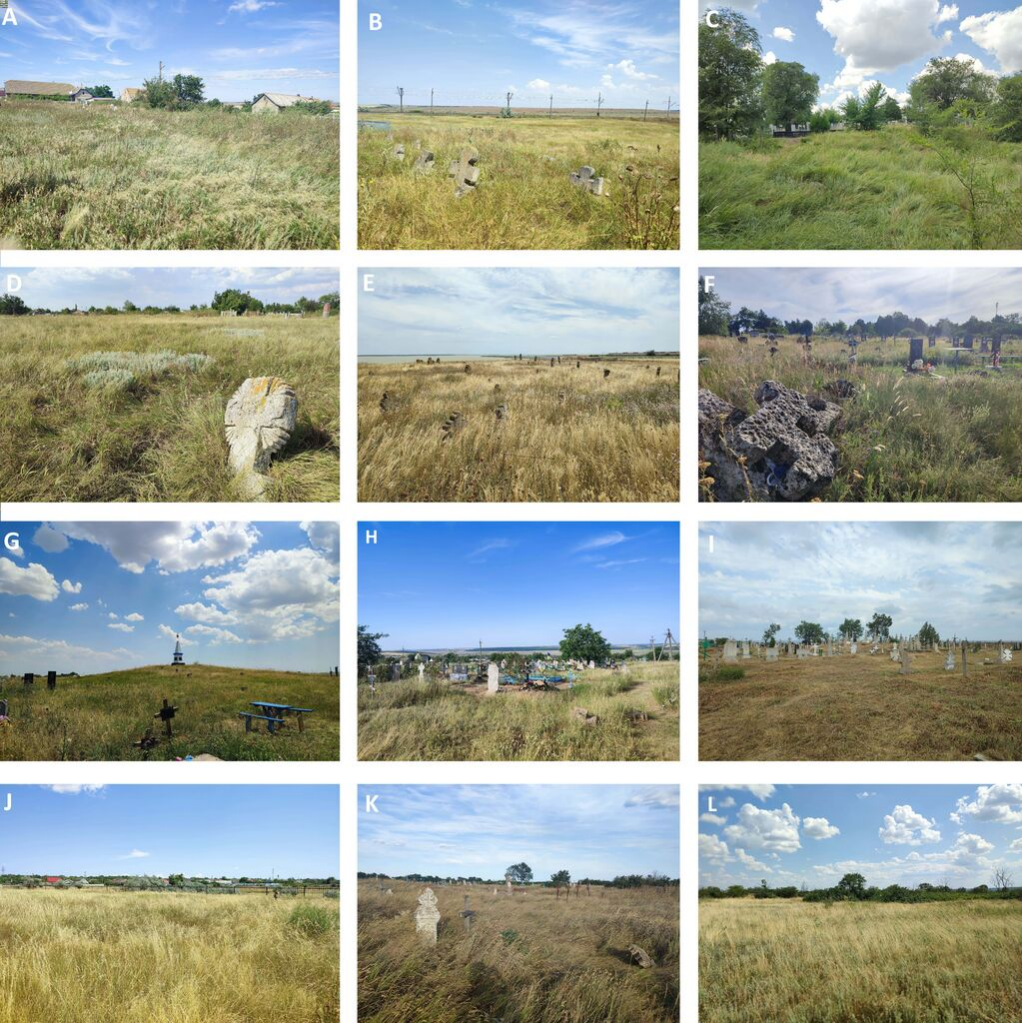
Examples of old cemeteries of the Right-Bank of Dnipro Grass Steppe. Abbreviations: **A** Borysivka; **B** Buldynka; **C** Chervonyi Tik; **D** Fedorivka; **E** Hlyboke; **F** Inhulka; **G** Kamianka; **H** Khrystoforivka; **I** Kosivka; **J** Kostiantynivka; **K** Lymany; **L** Shestirnia. Photos: N. Skobel.

**Figure 2.**
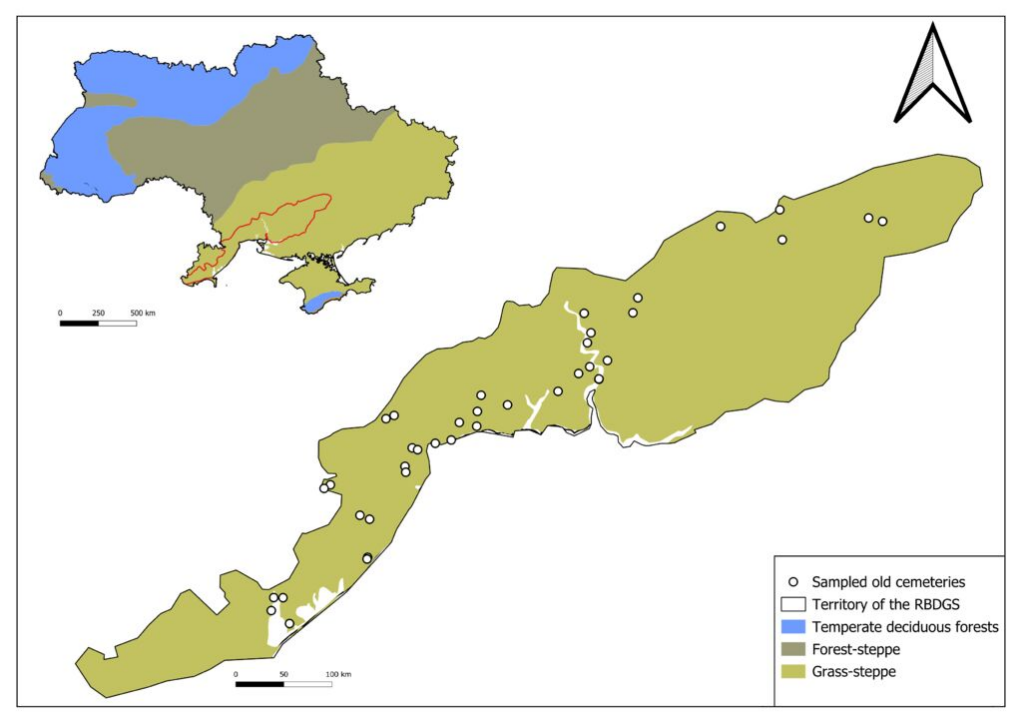
Map of Ukraine according to climatic definitions of the world’s terrestrial biomes (Loidi et al. 2022) and vector map (Loidi and Vynokurov 2024), with the study area -Right-Bank of Dnipro Grass Steppe (RBDGS), country border, extracted from OpenStreetMap (openstreetmap.org).

The area of the old cemeteries the varies from 0.1 ha to 32.5 ha. The total area of all old cemeteries is 112.91 ha (Figs 1, 2, Table 1).

### Sampling description

We searched for old cemeteries in the bibliographical sources for research. The selection of old cemeteries in 2023-2024 for the research was based on ongoing materials from the Google map "Ancient Cemeteries of Ukraine" (Ukraine Incognita 2025) and biographic data on the dates of foundation of human settlement community (Maps of Schubert 1865, Vasyliev 1967-1972,Malyna 2009). In the absence of precise dates, we used the founding date of the village as a necessary proximate estimate for the cemetery's establishment, as the two events typically occurred around the same time. The area and boundaries of the old cemeteries were determined using Google Earth and include the entire area of the old cemeteries, including the area of new burials if the old cemetery were extended (Google Earth 2025). We carried out a visual analysis, based on satellite images, with particular emphasis on the selection of cemeteries that were not overgrown and not covered with phanerophytes. In order to carry out a study of the flora of the area in question and of the presence of steppe refugia in the old cemeteries, we followed the following criteria, which could only be checked in the field:

● Presence of protected species (Red List of Dnipopetrovsk Region 2011, Red List of Mykovaliv Region 2012, Red List of Odesa Region 2013), Red Data Book of Ukraine (Didukh 2009);

● Presence of bunch-grasses (or tussock grasses such as *Festuca*, *Koeleria*, *Stipa* etc.) and other steppe species (from the class *Festuco*-*Brometea* (Mucina et al. 2016)).

To obtain this dataset, we investigated flora of 39 old cemeteries in the investigated area. To include different phenological aspects, we visited each site in different seasons of a year: in spring (April/May), summer (July) and autumn (October).

### Quality control

Some specimens of the vascular plants were collected and stored in the Herbarium of University of Warsaw (WA). After digitising the data, we harmonised the taxonomic information according to the nomenclature sources: for vascular plants “Ukrainian Plant Trait Database: UkrTrait v. 1.0” - (Vynokurov et al. 2024). Then we used GBIF Backbone Taxonomy (GBIF species matching tool: https://www.gbif.org/tools/species-lookup) for the taxonomic check and implemented minor corrections of species names regarding misprints and problematic taxa to avoid misinterpretation. We additionally checked and verified the header data using OpenRefine (https://openrefine.org/) and QGIS 3.38 (https://qgis.org) for quality control.

### Step description

1. Site selection, field research;
2. Identification of herbarium specimens of vascular plants;
3. Recording and identification of vascular plants with using road-field methods;
4. Digitising the field data forms;
5. Data checking and cleaning was performed using OpenRefine (OpenRefine 2025);
6. Transformation of the dataset according to the Darwin Core standards (Wieczorek et al. 2012);
7. Taxonomic check, final quality control.

## Geographic coverage

### Description

This area is situated in the south-western part of the Steppe Zone of Ukraine (Didukh and Shelyag-Sosonko 2003).

The Right-Bank Dnipro Grass Steppe District is characterised by low precipitation, which decreases from north to south and west to east (400-450 mm per year) (Marynych and Shyshchenko 2005). The average annual temperature exhibits a north-south gradient, with higher values observed in the southern regions and lower values in the northern regions (11.7°-8.4°C) (Karger et al. 2017). The average summer temperature is +30°C, the average winter temperature is no more than +4°C (Karger et al. 2017) with extreme temperatures of +40°C in summer and -28°C in winter (Karger et al. 2017). Soil resources of the Right-Bank Dnipro Grass Steppe District are represented by normal and southern chernozems and dark kastanozems in the southeast (Marynych and Shyshchenko 2005).

### Coordinates

47.96 and 44.80 Latitude; 34.80 and 28.96 Longitude.

## Taxonomic coverage

### Description

The Scientic Names of species are given, according to Ukrainian Plant Trait Database: UkrTrait v. 1.0 (Vynokurov et al. 2024). The total list of flora in situ includes 635 species of spontaneous vascular plants. All occurrences are classified to one phylum Tracheophyta, to three classes (Gnetopsida, Liliopsida, Magnoliopsida) and to 33 orders (Apiales, Asparagales, Asterales, Boraginales, Brassicales, Caryophyllales, Celastrales, Cornales, Cucurbitales, Dipsacales, Ephedrales, Ericales, Fabales, Fagales, Gentianales, Geraniales, Lamiales, Liliales, Malpighiales, Malvales, Myrtales, Oxalidales, Pinales, Piperales, Poales, Ranunculales, Rosales, Santalales, Sapindales, Saxifragales, Solanales, Vitales, Zygophyllales).

### Taxa included

**Table:** 

Rank	Scientific Name	
kingdom	Plantae	

## Temporal coverage

**Living time period:** 2023/2024.

## Usage licence

### Usage licence

Open Data Commons Attribution License

### IP rights notes

This work is licensed under a Creative Commons Attribution (CC-BY) 4.0 Licence.

## Data resources

### Data package title

Vascular plants of old cemeteries (southern Ukraine)

### Resource link

https://doi.org/10.15468/xbpxhj

### Number of data sets

1

### Data set 1.

#### Data set name

Records of vascular plants at the old cemeteries in the Right-Bank of Dnipro Grass Steppe District (southern Ukraine)

#### Data format

Darwin Core

#### Download URL

https://www.gbif.org/dataset/de0b1cd2-142e-4a22-961f-a2edc36a2ed9

#### Description

The dataset includes a table with 27 fields in Darwin Core terms and 6923 records (Skobel and Moysiyenko 2024).

**Data set 1.** 

Column label	Column description
occurrenceID	An identifier of a particular occurrence, unique within this dataset. We used the species occurrence numbers.
scientificName	The original names according to ‘Ukrainian Plant Trait Database: UkrTrait v. 1.0’, corrected for spelling mistakes using GBIF Species Matching tool.
eventDate	The date-time or interval during which an Event occurred.
basisOfRecord	The method in which data were acquired (HumanObservation).
geodeticDatum	The geodetic datum upon which the geographic coordinates are given. In our case, it is always WGS84.
georeferencedBy	The person who determined the georeference.
georeferenceProtocol	The description of the method used to determine coordinates (GPS).
recordedBy	The persons who is responsible for recording the original Occurrence.
identifiedBy	The persons who is responsible for recording the Taxon to the subject.
coordinateUncertaintyInMetres	The distance (in metres) from the given decimalLatitude and decimalLongitude describing the smallest circle containing the whole of the Location (from 40 m to 1100 m).
geoReferenceRemarks	Notes about the spatial description determination, explaining assumptions made in addition or opposition to those formalised in the method referred to in georeferenceProtocol (describing the smallest circle containing the whole of the Location (from 30 m to 1100 m).
decimalLatitude	The geographic latitude in decimal degrees.
decimalLongitude	The geographic longitude in decimal degrees.
organismQuantity	A number or enumeration value for the quantity of organisms. Estimated according to a 5-point scale: I - rare, 2 - occasional, 3 - frequent, 4 - common, 5 - abundant.
organismQuantityType	The type of quantification system used for the quantity of organisms. We used a 5-point scale.
continent	The name of the continent in which the Location occurs. In our case, it is always Europe.
countryCode	The standard code for the country in which the Location occurs. In our case, it is always UA.
country	The name of the country or major administrative unit in which the Location occurs. In our case, it is always Ukraine.
stateProvince	The name of the administrative region of Ukraine in which the Location occurs.
county	The full, unabbreviated name of the next smaller administrative region than stateProvince (districts).
locality	The specific description of the place. The nearest village and name of cemetery or official name of cemetery.
taxonRank	The taxonomic rank of the most specific name in the scientificName.
kingdom	The full scientific name of the kindom in which the taxon is classified. In our case, it is always *Plantae*.
phylum	The full scientific name of the phylum or division in which the taxon is classified. In our case, it is always *Tracheophyta*.
class	The full scientific name of the class in which the taxon is classified. In our case, it is *Magnoliopsida*, *Liliopsida*, *Gnetopsida*.
order	The full scientific name of the order in which the taxon is classified (Fig. 3; Taxonomic distribution of occurrences).
family	The full scientific name of the family in which the taxon is classified (Fig. 3; Taxonomic distribution of occurrences).

## Additional information

### Floristic richness and taxonomic value of old cemeteries

**Figure 3.**
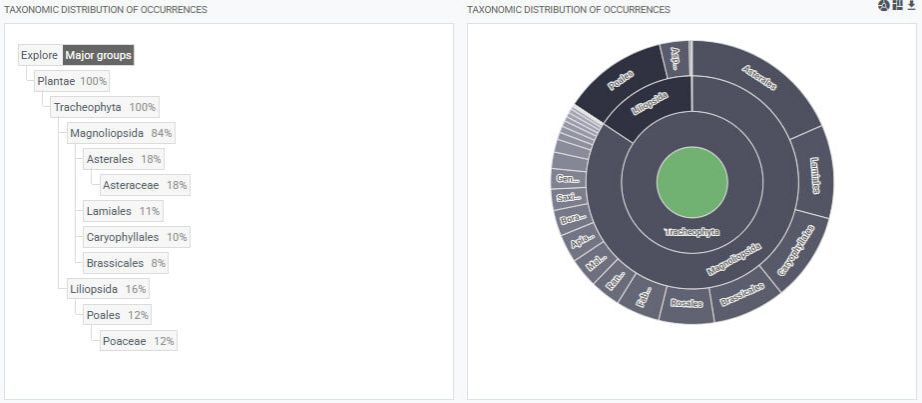
The taxonomic distribution of occurrences.

We identified 635 taxa of vascular plants in 39 old cemeteries, which make up 12.5% of the total flora of Ukraine (Mosyakin and Fedoronchuk 1999) and 31.4% of the flora of the northern Black Sea region (Moysiyenko 2013) and 69.3% Right-Bank Dnipro Grass Steppe District (Krytskaya 1985). Total species richness ranged from 84 (Pshonianove) to 242 (Nerubaiske). The mean number of vascular plant species per old cemeteries was 174. The majority of species (99.8%) belong to *Magnoliophyta* division (Fig. 3). The division Pinophyta (0.2%) is represented by one family – *Ephedraceae* and one species – *Ephedra distachya* L. The presence of *Lycopodiophyta*, *Polypodiophyta* and *Equisetophyta* in the flora of cemeteries was not confirmed, which is explained by unfavourable environmental conditions of the steppe zone (in particular, the insufficient level of moisture). A majority of 61.1% (388 species) of all species that occurred on the old cemeteries were native plants. Non-synanthropic plants occurring with the highest frequency were: *Asparagus officinalis*, *Centaurea borysthenica*, *Cephalaria uralensis*, *Convallaria majalis*, *Euphorbia seguieriana*, *Iris pumila*, *Kochia prostrata*, *Muscari neglectum*, *Paeonia tenuifolia*, *Phlomis tuberosa*, *Potentilla semilaciniosa*, *Salsola tragus*, *Sedum acre*, *Tanacetum millefolium* and *Viola ambigua*.

### Species-area relationships

Old cemeteries serve as refugia and biodiversity hotspots, exhibiting high floristic richness due to their long-term stability and high microhabitat heterogeneity compared to surrounding managed landscapes. Our analysis showed that the logarithmic model *S* = *c*+z*log(A) is the most statistically robust form of the Species-Area Relationship (SAR) for these data, which includes many small sites and a few extra-large ones. The model, with a stable slope (*z* ≈ 21.61), confirms a strong species saturation effect - species accumulation rapidly diminishes as area increases. This pattern indicates that the sites function close to their ecological carrying capacity and that the primary value for conservation lies in maintaining the stability and low-intensity management across the numerous small, established plots. Although four highly influential outlier sites were identified and removed, the core finding (*z* parameter) remained stable, confirming the robustness of the model and strengthening the conclusion that old cemetery fragments are reliable preserves of fitodiversity (Fig. 7). The model, while robust, operates within limitations imposed by the input data. The narrow range of habitat areas sampled in the core dataset restricts the generalisability of this model to significantly larger geographical scales.

### Conservation status of the species

We recorded 58 protected vascular plant species (10.4%), according to Resolution 6 of the Bern Convention (Council of Europe 1998), the Red Data Book of Ukraine (Didukh 2009) and Regional Red Lists of regions (Red List of Dnipopetrovsk Region 2011, Red List of Mykovaliv Region 2012, Red List of Odesa Region 2013):

— 3 species listed in the Resolution 6 of the Bern Convention (Council of Europe 1998) — *Iris hungarica*, *Jurinea cyanoides*, *Paeonia tenuifolia*.

— 15 species listed in the Red Data Book of Ukraine (Didukh 2009) — *Adonis vernalis*, *A. volgensis*, *Astragalus onobrychis*, *A. dasyanthus*, *Betula borysthenica*, *Cymbochasma borysthenica*, *Iris hungarica*, *Ornithogalum boucheanum*, *O. refractum*, *Paeonia tenuifolia*, *Stipa capillata*, *S. lessingiana*, *S. ucrainica*, *Tulipa biebersteiniana*, *T. schrenkii*.

— 24 species included in the Region al Red List of Dnipropetrovsk Region (Red List of Dnipopetrovsk Region 2011) — *Alcea pallida*, *Allium paniculatum*, *A. rotundum*, *Anemone sylvestris*, *Astragalus corniculatus*, *Convallaria majalis*, *Convolvulus lineatus*, *Dianthus guttatus*, *Ephedra distachya*, *Goniolimon besserianum*, *Haplophyllum suaveolens*, *Hesperis tristis*, *Inula oculus-christi*, *Iris pumila*, *Kohlrauschia prolifera*, *Linaria biebersteinii*, *Muscari neglectum*, *Ornithogalum kochii*, *Padus avium*, *Salvia austriaca*, *Sedum sexangulare*, *Sempervivum ruthenicum*, *Senecio borysthenicus*, *Thymus* x *dimorphus*, *Valeriana officinalis*.

— 9 species included in the Region al Red List of Mykolaiv Region (Red List of Mykovaliv Region 2012) — *Amygdalus nana*, *Anemone sylvestris*, *Astragalus pallescens*, *Convallaria majalis*, *Ephedra distachya*, *Iris pumila*, *Limonium platyphyllum*, *Polygonatum multiflorum*, *Sempervivum ruthenicum*.

— 15 species included in the Regional Red List of Odesa Region (Red List of Odesa Region 2013) — *Allium guttatum*, *Amygdalus nana*, *Anemone sylvestris*, *Arenaria leptoclados*, *Bellevalia sarmatica*, *Convallaria majalis*, *Dianthus lanceolatus*, *Helichrysum arenarium*, *Iris halophila*, *Iris pumila*, *Kohlrauschia prolifera*, *Muscari neglectum*, *Ornithogalum kochii*, *Phlomis hybrida*, *Valeriana officinalis*.

*Adonis vernalis*, *A. volgensis*, *Paeonia tenuifolia*, *Ornithogalum boucheanum*, *O. refractum*, *Tulipa biebersteiniana* and *T. schrenkii*, in old cemeteries, highlights the role of old cemeteries as refugia for protected vascular plant species in disturbed landscapes. The survival of species, characteristic of rapidly diminishing steppe ecosystems, is secured through a dual mechanism. Primarily, they represent relict populations in old cemeteries preserved in situ because the ground was historically protected from ploughing and intensive development. Secondly, these species are inadvertently supported by cultural practices and the aesthetic value of these plants. Some species (*Salvia nemorosa*, Thymus × dimorphus) often escape destruction during grave care because their attractive flowers are recognised and intentionally spared by families maintaining the plots .

Within the Orthodox tradition, the reverence for memory often necessitates active stewardship which manifests as the construction of enhancements and maintenance regimes involving weeding of graves, removal of undergrowth, planting of cultivated species and soil exposure. This human-mediated disturbance, while fulfilling cultural obligations, disrupts natural vegetation communities facilitating rapid colonisation by alien and invasive species and the subsequent loss of the native steppe flora (Skobel and Moysiyenko 2025). Conversely the limited availability of fiscal resources allocated for long-term management of aged cemeteries inadvertently promotes biodiversity because the resulting neglect maintains a low disturbance regime allowing for the re-establishment and stabilisation of the native steppe vegetation. This situation creates a paradox where active care reduces native biodiversity through ecosystem disruption, while minimal intervention and neglect preserves the integrity of remnant steppe communities.

**Figure 4.**
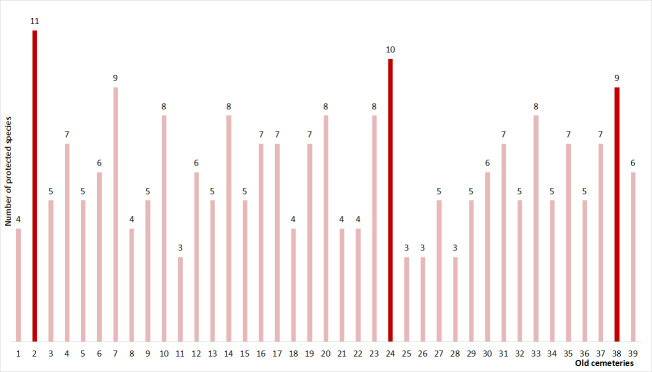
The total number of protected species in the floristic composition for each old cemetery. Dark red colour represents the three highest values. Abbreviations: 1 — Balovne, 2 — Borysivka, 3 — Buldynka, 4 — Velyka Korenykha, 5 — Velykyi Dalnyk, 6 — Vypasne, 7 — Hlyboke, 8 — Jewish cemetery c. Bilhorod-Dnistrovskyi, 9 — Yelysavetivka, 10 — Inhulka, 11 — Kamianka, 12 — Kozatske, 13 — Korolivske, 14 — Kosivka, 15 — Kostiantynivka, 16 — Kryzhanivka, 17 — Lymany, 18 — Liubopil, 19 — Mykolaivskyi Nekropol, 20 — Nerubaiske, 21 — Nova Dofynivka, 22 — Novobohdanivka (big cemetery), 23 — Novobohdanivka (small cemetery), 24 — Odradove, 25 — Popazdra, 26 — Prylymanske, 27 — Pshonianove, 28 — Sebyne, 29 — Sychavka, 30 — Skobeleve, 31 — Starokozachie, 32 — Trapivka, 33 — Usatove, 34 — Ust-Kamianka, 35 — Fedorivka, 36 — Khrystoforivka, 37 — Chervonyi Tik, 38 — Shestirnia, 39 — Shyroke.

**Figure 5.**
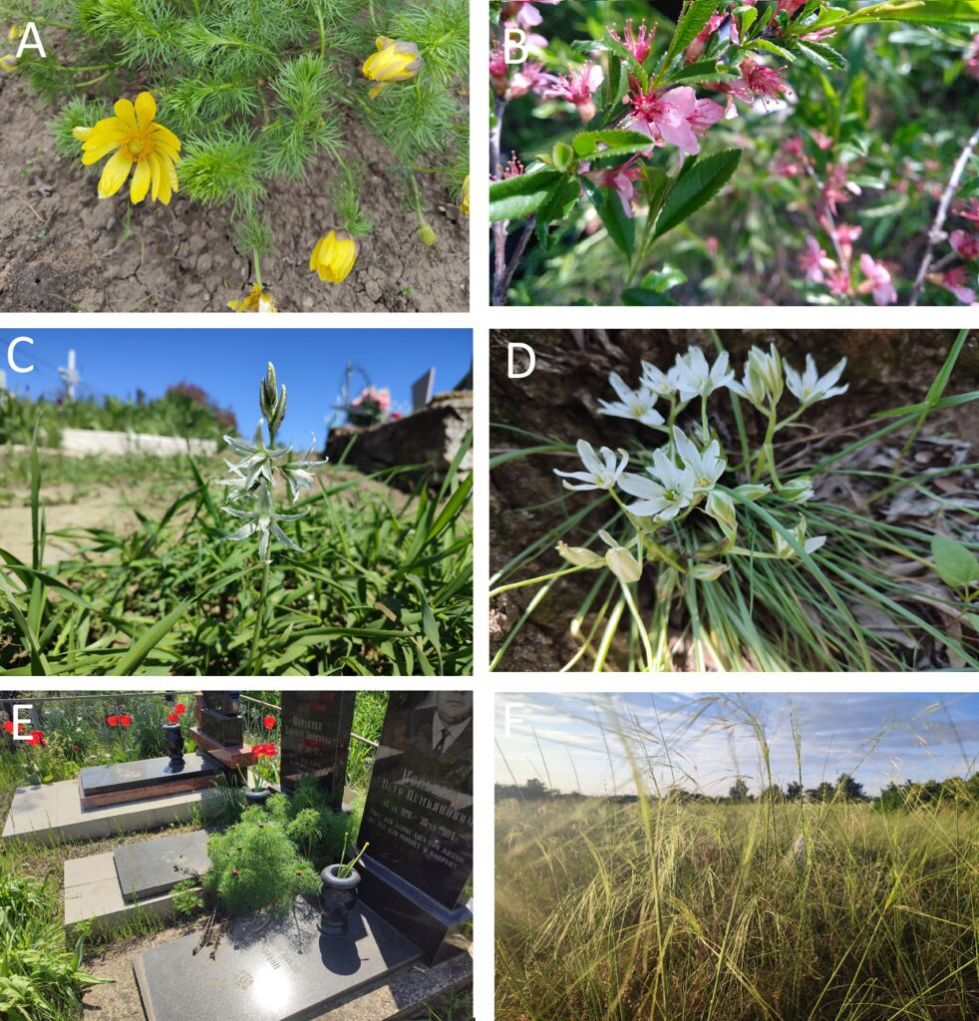
Examples of protected species of the Right-Bank Dnipro Grass Steppe. Abbreviations: **A** *Adonis vernalis*; **B** *Amydalus nana*; **C** *Ornithogalum boucheanum*; **D** *O. refractum*; **E** *Paeonia tenuifolia*; **F** *Stipa capillata*. Photos by N. Skobel.

**Figure 6.**
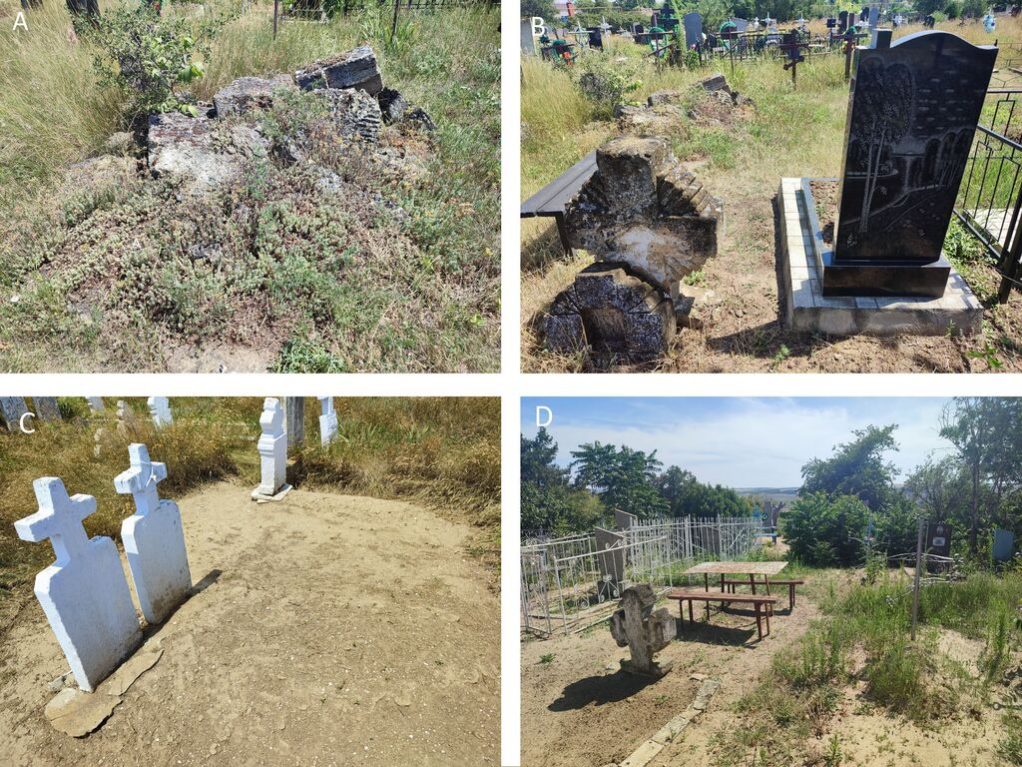
Types of anthropogenic pressure within the old cemeteries of the Right-Bank of Dnipro Grass Steppe District. Abbreviations: **А, B** reburials; **C, D** weeding grave. Photos: N. Skobel.

**Figure 7.**
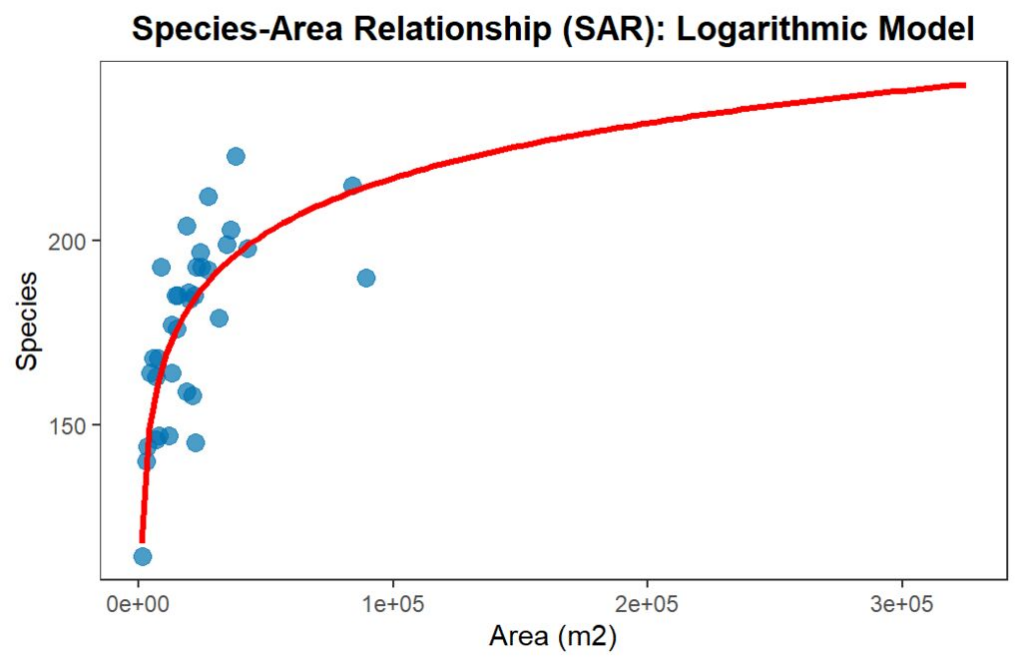
Relationship between Floristic Richness (Species) and Habitat Area (m^2^) in Old Cemeteries. The data are fitted with a Logarithmic Linear Model.

**Old cemeteries in Ukraine are characterised by a number of challenges** when it comes to preserving biodiversity (Moysiyenko et al. 2021, Skobel and Moysiyenko 2021, Skobel et al. 2023, Skobel and Moysiyenko 2024, Skobel et al. 2024, Skobel and Moysiyenko 2025). These include elimination of sites, re-purposing of land, reburials and the absence of a management plan (Figs 4, 5, 6). A salient issue is the absence of a comprehensive register of old cemeteries. This lack of documentation has the potential to result in the neglect and subsequent destruction of many historic burial grounds. The transformation of biologically valuable old cemeteries into formally recognised Nature Conservation Areas represents a critical administrative and legal step to guarantee the long-term protection of their flora. Achieving conservation status requires a coordinated administrative effort utilising national and international legal instruments for effective, long-term stewardship.

### Potential of design and conservation of the Emerald Network territories within old cemeteries in southern Ukraine

It is suggested that active conservation intervention in natural burial sites should only take place if the protection of the site cannot be ensured otherwise. This strategy is also favourable in view of the limited capacity of policy-makers to manage conservation issues (Löki et al. 2019).

It is important to note that there is a movement in Ukraine aimed at preserving the historical and cultural heritage of old cemeteries, implemented by the NGO, Ukraine Incognita, within the framework of the "Ancient Cemeteries of Ukraine" project (Ukraine Incognita 2025). In the future, for some particularly valuable sites, it will be possible to introduce comprehensive protection that will contribute to the preservation of natural, historical and cultural values. A similar approach to cultural heritage sites in Ukraine has already been proposed for burial mounds (Sudnik-Wójcikowska and Moysiyenko 2012).

The presence of species listed in the Red Book of Ukraine and regional Red Lists provides a basis (according to current Ukrainian law) for the creation of nature conservation areas (Verkhovna Rada Ukrainy 1992). In our opinion, old abandoned cemeteries could be designated as natural monuments. The designation of natural monuments is carried out without expropriating land plots and other natural objects from their owners. Natural monuments are individual unique natural formations with special environmental, scientific, aesthetic, educational and cultural significance. Their designation aims to preserve them in their natural state (Verkhovna Rada Ukrainy 1992).

The old cemeteries contain three types of protected habitats from Resolution 4 of the Bern Convention: E1.2 Perennial calcareous grasslands and basic steppes, F3.241 Central European subcontinental thickets and F3.247 Ponto-Sarmatic deciduous thickets. The habitat E1.2 is characterised by the highest conservation value (Skobel et al. 2024).

Moreover, the presence of species listed in Resolution 6 of the Bern Convention (Council of Europe 1998) and habitats listed in Resolution 4 of the Bern Convention (Council of Europe 1996) provides a rationale for establishing an Emerald Network site within old cemeteries – to protect these sites from new burials and reburials and to support the implementation of a management plan.

The lack of management plans has resulted in the accumulation of litter in most of these cemeteries. We propose to preserve the steppe flora of old cemeteries through orderly management. In particular, mowing and cutting of tree and shrub vegetation would be most appropriate. We have observed grazing and burning in some old cemeteries, but we do not consider these practices ethical to recommend as management recommendations at these cultural heritage sites.

For Ukraine, the establishment of the Emerald Network, as a component of the pan-European ecological network, represents a key element of the European integration process. This initiative is being implemented within the framework of the provisions of the Convention on the Conservation of European Wildlife and Natural Habitats (Council of Europe 1978), which has been ratified by Ukraine. The integration of Emerald Network territories into the Natura 2000 network is expected upon the country's accession to the European Union, given that both networks are designed according to similar principles (Vasyliuk et al. 2019).

According to the Conservation Evidence, the action "Legally protected habitats" is likely to be beneficial (Walsh et al. 2023). The establishment and development of local and European protected areas in Ukraine is undoubtedly valuable for conservation. Such protection will help ensure that old cemeteries are preserved and not destroyed. The purpose of a conservation management plan is to implement the conservation strategy and to establish detailed objectives for the integrated management of natural and cultural heritage sites. It is an important tool widely used in many countries to guide future care and sustainable use.

### Conclusion

Old cemeteries, persisting within the intensively cultivated landscape of Ukraine, constitute unique enclaves of endangered and protected vascular plant species. Together with other remaining fragments of natural or semi-natural steppe vegetation, they could play a significant role in the restoration of European steppes. The creation of legally mandated protected areas is essential. This formal administrative designation provides the legal mandate to shield these conservation assets from detrimental anthropogenic processes (liquidation, land re-profiling and repeated disruptive burials), ensuring the long-term in situ persistence of their natural heritage.

## Figures and Tables

**Table 1.** Overview of the investigated old cemeteries in the area of the Right-Bank of Dnipro Grass Steppe.

№	**Site location**	**Region**	**L atitude**	**L ongitude**	The establishment date of the village (*date of the earliest probable burial)	**Area [ha]**
1	Balovne	Mykolaiv	47.05375	31.88997	1679	2.29
2	Borysivka	Odesa	45.79252	29.63721	1860th	2.00
3	Buldynka	Odesa	46.66259	30.97027	1803	1.88
4	Chervonyi Tik	Dnipropetrovsk	47.66093	33.91062	1927	1.50
5	Fedorivka	Mykolaiv	46.74904	31.31671	1800	1.32
6	Hlyboke	Odesa	45.72758	29.61954	1841	2.73
7	Inhulka	Mykolaiv	47.20028	32.21721	1802	4.26
8	Jewish cemetery c. Bilhorod-Dnistrovskyi	Odesa	46.18348	30.32564	1855-1865	0.35
9	Kamianka	Mykolaiv	46.81579	31.67946	1790	2.41
10	Khrystoforivka	Mykolaiv	47.27312	32.25278	1799	1.20
11	Korolivske	Mykolaiv	46.90317	31.82694	1926 (*1860th)	0.31
12	Kosivka	Odesa	45.99369	30.31176	1834	0.70
13	Kostiantynivka	Mykolaiv	47.10246	31.91573	1783	2.46
14	Kozatske	Odesa	46.35441	30.04403	1774	3.14
15	Kryzhanivka	Odesa	46.55970	30.79762	1775	0.75
16	Liubopil	Odesa	46.71708	31.10016	1886	1.88
17	Lymany	Odesa	45.66227	29.75072	1812	3.62
18	Mykolaivskyi Nekropol	Mykolaiv	46.96710	32.03437	1795	32.5
19	Nerubaiske	Odesa	46.53696	30.63211	1795	4.67
20	Nova Dofynivka	Odesa	46.57588	30.91226	1860th	3.83
21	Novobohdanivka (large cemetery)	Mykolaiv	46.87475	31.97262	1865	1.47
22	Novobohdanivka (small cemetery)	Mykolaiv	46.87691	31.97300	1920th	0.70
23	Odradove	Odesa	46.68112	30.44364	1637	0.87
24	Popazdra	Odesa	45.98527	30.30772	1824	0.81
25	Prylymanske	Odesa	46.41669	30.58563	1793	0.17
26	Pshonianove	Odesa	46.79646	31.12706	1850	0.10
27	Sebyne	Mykolaiv	47.19786	31.86705	1792	0.89
28	Shestirnia	Dnipropetrovsk	47.55568	33.29056	1689	2.25
29	Shyroke	Dnipropetrovsk	47.70054	33.27255	1787	8.94
30	Skobeleve	Mykolaiv	47.62009	32.84738	1875	1.55
31	Starokozachie	Odesa	46.33639	29.99886	1824	3.48
32	Sychavka	Odesa	46.64408	31.09515	1801	1.95
33	Trapivka	Odesa	45.79188	29.70363	1829	2.12
34	Usatove	Odesa	46.52809	30.67081	1775	8.40
35	Ust-Kamianka	Dnipropetrovsk	47.64458	34.01116	1754	0.58
36	Velyka Korenykha	Mykolaiv	46.93656	31.90702	1860th	0.34
37	Velykyi Dalnyk	Odesa	46.44588	30.57997	1795	2.72
38	Vypasne	Odesa	46.20309	30.25639	1795	1.32
39	Yelysavetivka	Odesa	46.69705	30.50187	1856	0.45
